# Angiosarcoma of Small Bowel Presenting with Obstruction: Novel Observations on a Rare Diagnostic Entity with Unique Clinical Presentation

**DOI:** 10.1155/2012/480135

**Published:** 2012-10-21

**Authors:** Richard Siderits, Frederick Poblete, Biren Saraiya, Cheryl Rimmer, Anup Hazra, Le Aye

**Affiliations:** ^1^Robert Wood Johnson University Medical School, Piscataway, NJ 08854, USA; ^2^Department of Surgery, General Surgery Service, RWJ University Hospital Hamilton, Hamilton, NJ 08690, USA; ^3^Cancer Institute of New Jersey at Hamilton, Medical Oncology Service, Hamilton, NJ 08690, USA; ^4^New Jersey School of Osteopathic Medicine, University of Medicine and Dentistry, Stratford, NJ 08084, USA

## Abstract

We present a case of angiosarcoma in small bowel, presenting with partial small bowel obstruction in a 79-year-old man with no history of radiation, chemotherapy, toxin exposure, or previous operative intervention. Angiosarcoma of small bowel is a rare entity which may present with nausea, abdominal pain, recurrent bleeding, and usually a history of prior radiation or exposure to specific toxins (polyvinyl chloride). Angiosarcoma of small bowel tends to spread rapidly and has a poor prognosis. We review the surgical and oncologic challenges. We report unique macroscopic findings of raised hyperemic margins, which are suggestive of a vasogenic lesion and the histologic feature of a partially retiform pattern with dense basement membrane material in an otherwise poorly differentiated lesion.

## 1. Introduction

Small bowel tumors of any type are infrequent, and the differential diagnostic evaluation at presentation has many challenges [1]. Angiosarcoma of small bowel is an exceedingly rare entity and demonstrates rapid dissemination and a correspondingly poor prognosis [2]. Presenting symptoms for angiosarcoma of small bowel may be nonspecific and for most of the few reported cases include recurrent gastrointestinal bleeding, abdominal pain, and nausea; however, small bowel (ileal-ileal) intussusception has been also documented [3, 4]. Angiosarcoma of small bowel may be associated with previous radiation treatment, chemotherapy, and chemical toxin exposure, specifically polyvinyl chloride [4]. Excluding cutaneous sites, the organs most often involved as primary sites are heart, liver, and spleen [5, 6]. Metastatic angiosarcoma from a hepatic primary has also documented [7]. 

We describe the clinical and pathologic features, as well as the differential diagnosis, of a rare case of angiosarcoma of small bowel in a 79-year-old man with no prior history of intra-abdominal radiation who presented with small bowel obstruction. 

## 2. Clinical Presentation and Clinical Course

**Figure 1:**
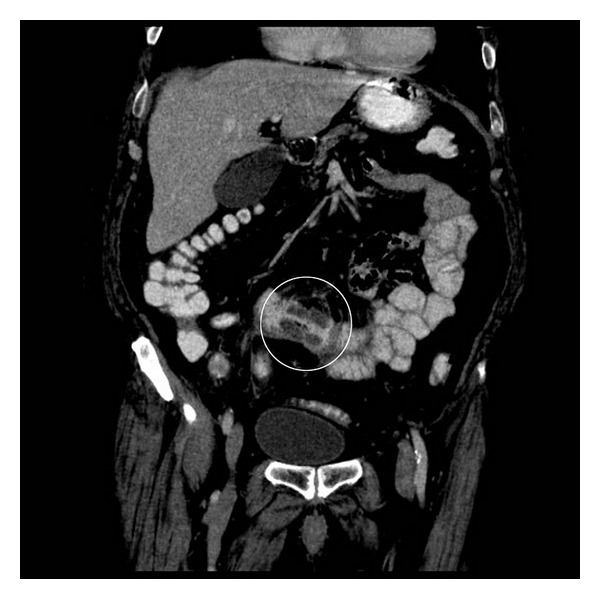
Apple core lesion in small bowel, see circle (computed tomography).

A 79-year-old male with no prior history of intra-abdominal radiation or environmental exposure to polyvinyl chloride presented to the emergency department with a complaint of diarrhea for seven days and diffuse abdominal pain that started the previous day. On admission, he complained of diffuse lower abdominal pain. Laboratory evaluation revealed white blood cell count of 15 × 10^3^/mm^3^. A computed tomography (CT) scan of the abdomen and pelvis with contrast showed air filled dilated loops of small bowel with scattered air-fluid levels felt to represent an early obstruction. The admission diagnosis was partial small bowel obstruction. After the initial 2 days of the hospital stay, the patient appeared to improve clinically with decrease in white blood cell count, decrease abdominal pain while tolerating an oral diet; however, a repeat CT scan now revealed an apple core lesion near the mid jejunum (see Figure 1). An exploratory laparoscopy was performed and a portion of mid jejunum was resected. The procedure lasted 30 minutes with minimum blood loss (approximately 10 cc). The post-operative period was uneventful. The patient was discharged home 3 days after the surgery and was instructed to follow up with oncology. 

**Figure 2:**
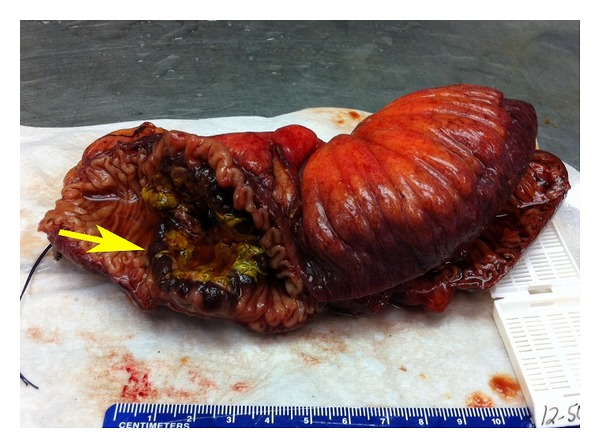
Surgical specimen of the resected small bowel.

The surgical specimen consisted of a segment of small bowel measuring 21 cm in length. The attached connective tissue appeared hemorrhagic. The specimen was opened to reveal an exophytic tumor measuring 4.2 cm in greatest cross dimension with ulcerated and hemorrhagic base. The lesion was oriented circumferentially with almost complete obstruction of bowel lumen. Interestingly, the raised margins of the lesion appeared markedly hyperemic. We feel that this is unique to this type of vascular tumor and may serve as a differentiator from adenocarcinoma or carcinoid tumors of small bowel. Surgical margins of the specimen were viable and free of involvement (see Figure 2).

**Figure 3:**
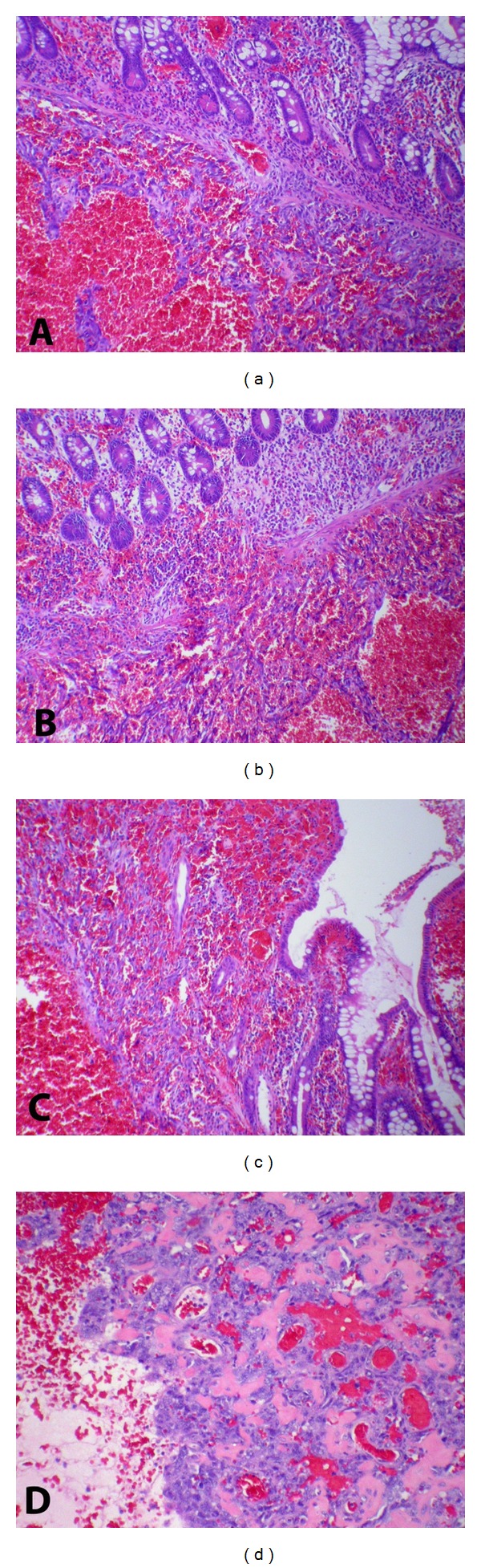
(a) Tumor underneath muscularis mucosa of small bowel; (b) edge of tumor with disruption of muscularis mucosa; (c) tumor extending into mucosa at edge of tumor (see macroscopic image Figure 1); (d) portion of tumor showing retiform “hemangioepithelioma.”

**Figure 4:**
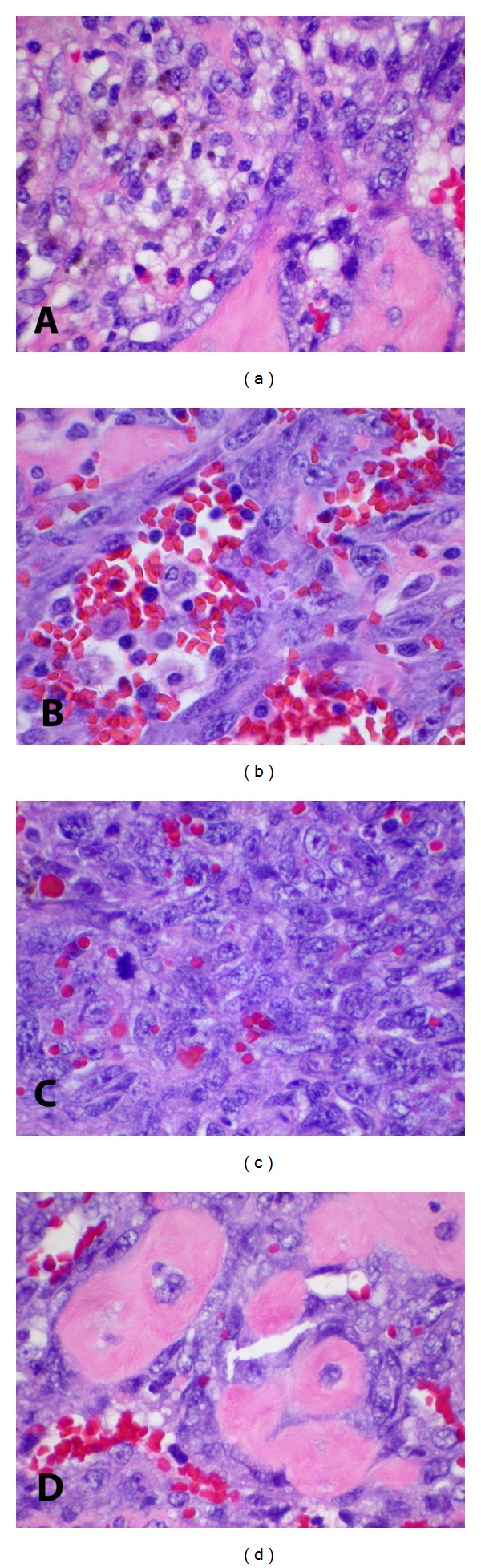
(a) Hemosiderin deposition in tumor; (b) anastomosing vascular channels; (c) frequent atypical mitotic figures and intra-cytoplasmic “vascular channels;” (d) dense collagen deposition in basement membrane type pattern.

The histologic diagnosis was that of angiosarcoma of small bowel arising in submucosa, with transgression of muscularis propria at radial surgical margins of tumor. The proliferative rate, as estimated by mitotic figures, was high. Tumor showed predominantly spindle-shaped cells without significant epithelioid features. These cells showed marked nuclear pleomorphism and occasional intracytoplasmic lumen. There were no eosinophilic hyaline bodies identified. The tumor extended from the submucosa to the areas corresponding to the hyperemic tumor edges, providing the uniquely dark red appearance of the tumor margin (see Figures 3 and 4). Immunohistochemical evaluation reveals the followings: strongly positive for CD31 and negative for EMA, CD117, SMA, AE1/AE3, and S100. Human Herpes Virus 8 (HHV-8) was negative.

## 3. Discussion

Angiosarcoma is rare (representing 1-2% of soft tissue sarcomas) and most often arises in subcutis. Involvement of internal organs is predominantly heart, liver, and spleen [5, 6]. The exact pathophysiologic process of angiosarcoma is unclear, but the association with chemotherapy, chemical toxins (polyvinyl chloride), and external beam radiation has been described. 

We report a case of primary angiosarcoma of small bowel in a 79-year-old man who presents with partial small bowel obstruction. Presenting signs and symptoms for this rare entity, as reported in the medical literature, most often include abdominal pain, nausea, recurrent gastrointestinal bleeding, however, obstruction without intussusception or prior irradiation has not been documented. This tumor tends to spread rapidly and has a more advanced stage at diagnosis; therefore, prognosis is grim. 

In our review of English literature, most patients with angiosarcoma of small bowel presented with abdominal pain, nausea, and diarrhea. In addition, these patients may require frequent blood transfusions, and death often results from uncontrollable hemorrhage [8]. There are only two cases of angiosarcoma of small bowel which presented as small bowel obstruction and both patients had prior history of radiation [9]. To our knowledge, our patient is the first case to present with small bowel obstruction without prior history of either radiation or intussusception. Our patient had only minimal blood loss during the surgery, and there were no signs of GI bleeding. 

Angiosarcoma may show features similar to spindle cell melanoma, spindle cell carcinoma, and mesothelioma. The cells stain for vascular markers such as CD 31 (90% angiosarcomas), CD34 (50% of angiosarcoma), and less often with von Willebrand factor and occasionally show cytokeratin immunoreactivity [10]. CD 31 is the most reliable vascular tumor marker for the diagnosis of angiosarcoma [11]. The D2-40 antibody is positive in angiosarcoma, lymphangiosarcoma, and Kaposi's sarcoma; therefore, it is not useful in differentiation [12]. HHV-8 was negative in this case, favoring the diagnosis of angiosarcoma over Kaposi's sarcoma [3, 13]. 

Macroscopic findings generally include a markedly hemorrhagic appearance with poorly defined invasive margins. The histologic differentiation may vary from benign appearing vascular proliferation to undifferentiated malignancy. The histologic presentation includes vascular abnormalities of the small intestines which may present as florid vascular proliferation. These lesions show minimal nuclear atypia and low mitotic rate [14]. 

Differential diagnostic considerations include hemangioma, Kaposi's sarcoma (especially predominant spindle cells type), lymphangiosarcoma, poorly differentiated carcinoma, or amelanotic spindle cell variant of melanoma. Among the three stages of Kaposi sarcoma (KS), the advanced patch stage of Kaposi sarcoma resembles the well-differentiated angiosarcoma. The histologic features that help differentiate angiosarcoma from KS are the presence of cavernous vessels and epitheloid endothelial cells seen in angiosarcoma and the presence of intra- and extracellular eosinophilic hyaline granules in KS [15]. In addition, KS demonstrates positive for smooth muscle actin (SMA) staining (>95%) whereas angiosarcoma does not [15]. Commercially available antibodies against latency-associated nuclear antigen (LANA-1) encoded by the HHV8 are highly sensitive and specific for diagnosing Kaposi sarcoma. Histologically, lymphangiosarcoma can be very similar to angiosarcoma. Perhaps, the distinctive feature of lymphangiosarcoma is its association with lymphangiomatosis. The vessels are dilated, diffuse throughout the soft tissue and lined by plump endothelial cells with hyperchromatic nuclei [16]. 

Since the tumor of our surgical specimen is strongly positive for CD31, it confirms our diagnosis of a vasogenic lesion. The tumor is negative for S100, SMA, and CD117 and helps to exclude melanoma, Kaposi sarcoma, and an epitheloid gastrointestinal stromal tumors. 

The clinical course, for most well-documented sites, includes repeated local recurrences. Angiosarcomas tend to spread by hematogenous mechanism and the most frequent metastatic sites depend on site of origin. Angiosarcomas of small bowel tend to metastasize to liver, lung, lymph node, other intra-abdominal sites, skin, spleen, urinary bladder, bone, gall-bladder, pancreas, and brain. Although rare, patients with angiosarcoma of the gastrointestinal tract have a very aggressive clinical course with a 6-month prognosis following initial diagnosis. Death is most often related to refractory bleeding and metastasis [17]. Treatment options include palliative surgical resection (since complete resection often not possible due to infiltrative nature of the tumor). Given the rarity of the angiosarcoma of small intestines, there are no large trials that provide guidance for systemic therapy. Studies of soft tissue sarcoma suggest paclitaxel (weekly or every three weeks), doxorubicin, docetaxel, and liposomal doxorubicin may have benefit [18]. There are reports of complete responses and partial responses with these regimens [19]. 

The French Sarcoma Group recently published a phase II study of targeted therapy with Sorafenib, a small molecule inhibitor of VEGFR and B-RAF in cutaneous and visceral angiosarcoma [19]. The rationale was based on the findings of recent studies showing presence of overexpression of mRNA and proteins of vascular growth factors and receptors in angiosarcoma cell lines that include vascular endothelial growth factor (VEGF)-A, VEGF-C, VEGF receptor (VEGF-R1), VEGF-R3, vascular permeability factor (VPF), FLT-A, KDR (VEGF-R2), and v-ets erythroblastosis virus E26 oncogene homolog 1 (ETS-1) [19]. While the study did not meet objective of progression free survival of 9 months, it is the first study that evaluates therapy based on the potential pathophysiology of the angiosarcoma. 

Staging follows the American Joint Cancer Committee format for visceral sarcomas of small intestine. A tumor perforating visceral peritoneum as seen in the present case should be staged as T4. Lymph node metastasis in soft tissue sarcomas is reported as N1; and presence of positive nodes (N1) in M0 tumors is considered Stage III. Whereas metastasis in 1 to 3 regional lymph nodes is N1, and metastasis in 4 or more regional lymph nodes is N2. 

In summary, we report unique macroscopic features of a small bowel angiosarcoma (presenting as obstruction) in an elderly male patient without either recurrent gastrointestinal bleeding, history of prior irradiation, or specific toxin exposure. This is not only a rare diagnostic entity in itself but also, we believe, a novel presentation of obstruction without a history of either prior radiation or toxin exposure. This entity usually has an aggressive course with a poor prognosis. This entity should be in the differential diagnosis or be a diagnostic consideration in a patient who presents with obstruction and a tumor with raised markedly hyperemic tumor margins.
